# Outcomes of a regional suicide prevention systems intervention study: Suicide prevention by monitoring and collaborative care (SUPREMOCOL) in noord-brabant in the netherlands

**DOI:** 10.1192/j.eurpsy.2021.471

**Published:** 2021-08-13

**Authors:** C. Van Der Feltz-Cornelis

**Affiliations:** Health Sciences, University of York, Heslington, York, United Kingdom

**Keywords:** SUPREMOCOL, Digital monitoring, Suicide prevention, Systems intervention

## Abstract

**Introduction:**

Since 2007, suicide rates increased in the Netherlands and the province of Noord-Brabant ranked second nationally with a 64% increase. 60% of people who died by suicide did not receive treatment at the time of their death. Gap analysis showed 1) lack of expertise to explore suicide risk in health care or community settings where persons at risk presented; 2) lack of swift access to specialist care addressing suicidality; 3) lack of oversight of the care process and 4) lack of follow up.

**Objectives:**

We developed a regional suicide prevention systems intervention with chain partners at community, general health and mental health care level to address these gaps in Noord-Brabant, aiming at a 20% decrease in the number of suicides.

**Methods:**

The project started October 2016 and lasted 4 years. The intervention has four pillars: 1) Online decision aid for health care professionals to assess suicidal risk and to communicate with chain partners; 2) swift access to care; 3) facilitation of care through the care chain by a dedicated nurse; and 4) 12 months follow up monitoring if the patient still receives appropriate care. We examined the effect of SUPREMOCOL on suicides in a pre-post design.

**Results:**

During the implementation year of the intervention, suicides in Noord-Brabant dropped 17% whereas nationally they dropped 5%, and this effect was sustained after one year.

**Conclusions:**

This suicide prevention systems intervention is effective in reducing suicide rates. Long-term follow-up and implementation is warranted.

**Disclosure:**

No significant relationships.

